# Men’s preconception diet quality patterns predict supportive food parenting practices: evidence from a longitudinal cohort study

**DOI:** 10.1186/s12966-026-01914-z

**Published:** 2026-05-01

**Authors:** Mariane H. De Oliveira, Brian K. Lo, Matthew M. Lee, Stephanie Armbruster, Natalie Grafft, In Young Park, Alejandra Cantu-Aldana, Katherine W. Bauer, Rebekah Levine Coley, Sebastien Haneuse, Kirsten Davison, Jess Haines

**Affiliations:** 1 Boston College, School of Social Work, Chestnut Hill, MA USA; 2 Department of Family Relations & Applied Nutrition, University of Guelph, Guelph, ON Canada; 3 Harvard T.H. Chan School of Public Health, Boston, MA USA; 4 Chungnam National University, Daejeon, Republic of Korea; 5 Division of Human Genetics, The University of Texas Rio Grande Valley, Brownsville, TX USA; 6 Department of Nutritional Sciences, School of Public Health, University of Michigan, Ann Arbor, MI USA; 7 Boston College, Lynch School of Education and Human Development, Chestnut Hill, MA USA

**Keywords:** Fathers, Food parenting practices, Children, Dietary patterns, Healthy eating index, Cohort study

## Abstract

**Background:**

Food parenting practices play a vital role in shaping children’s food intake, yet evidence linking fathers’ earlier diet patterns to later parenting is limited. This study examined the association between fathers’ diet quality patterns during their adolescence and food parenting practices during fatherhood.

**Methods:**

Data were drawn from Fathers & Families (F&F), a father-based cohort that recruited participants from an ongoing cohort in the United States that has followed participants since adolescence. Participants (*n* = 584) reported their dietary intake during adolescence (ages 10–18) using a Youth/Adolescent Food Frequency Questionnaire across multiple survey waves (1996–2011), and reported their food parenting practices using an online survey completed in 2021–2022. Fathers’ diet quality patterns during their adolescence were derived from Healthy Eating Index-2020 (HEI-2020) scores using sequence analysis and hierarchical clustering. Associations between these adolescent diet quality patterns and food parenting practices (coercive control, structure, autonomy support) were estimated with ordinal logistic regression, adjusting for sociodemographic covariates and family meal frequency measured during adolescence.

**Results:**

Three diet quality patterns were identified in adolescence: Low HEI-2020 (50.0%), Declining HEI-2020 (36.5%), and Increasing HEI-2020 (13.5%). Compared to those with Low HEI-2020, fathers demonstrating Increasing HEI-2020 had higher odds of using supportive food parenting practices with their preschool-aged children including higher use of structure-based food parenting practices (OR = 1.93, 95%CI [1.18–3.18]) and lower use of coercive control-based food parenting practices (OR = 0.57, 95%CI [0.36–0.91]).

**Conclusions:**

Improving men's diet quality during adolescence may have enduring benefits, promoting not only healthier adult eating patterns but also more supportive food parenting practices as fathers.

**Graphical Abstract:**

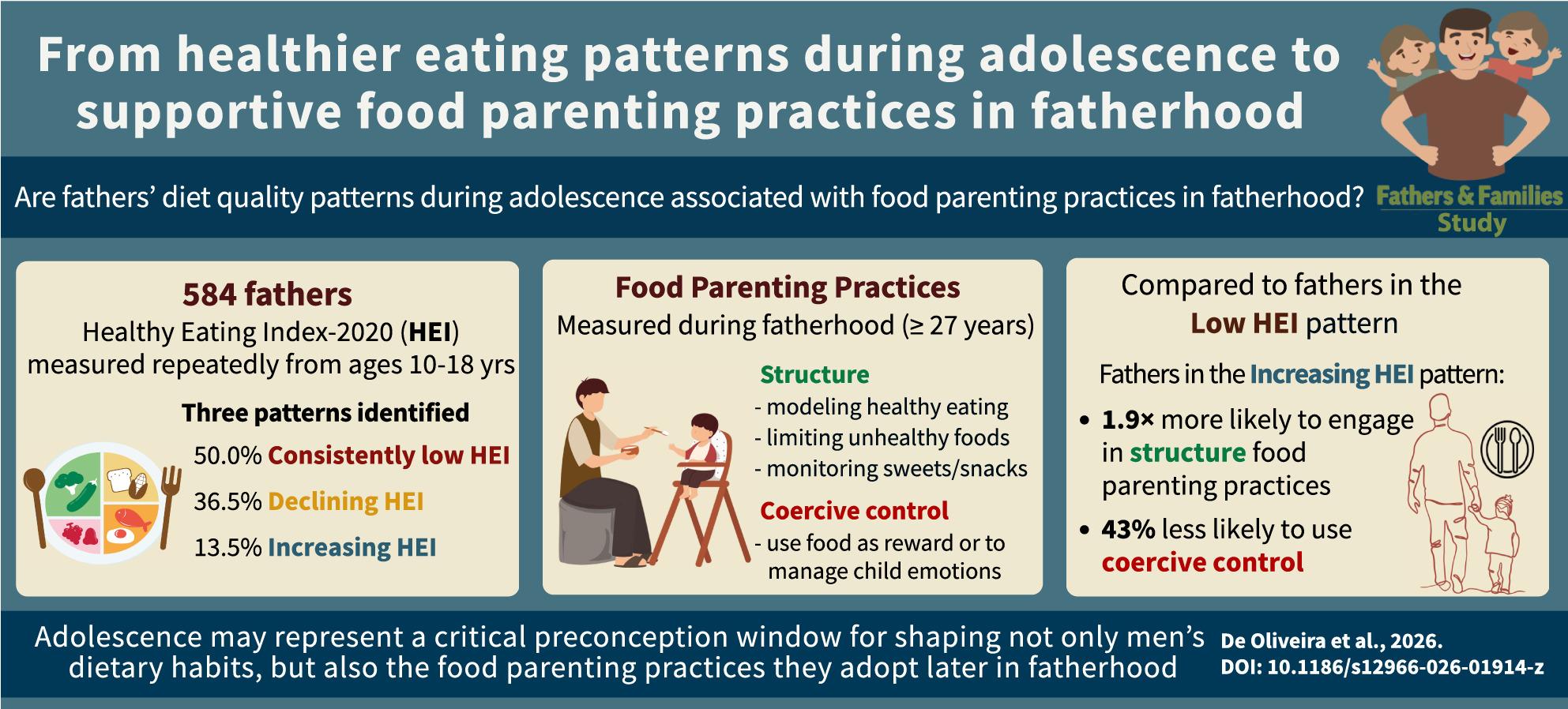

**Supplementary Information:**

The online version contains supplementary material available at 10.1186/s12966-026-01914-z.

## Background

Food parenting practices refers to the approaches and actions parents take to shape their children’s eating behaviors [1]. These practices are typically categorized into three domains: coercive control (e.g., giving children food when upset or bored, using sweets as a reward or punishment), structure (e.g., keeping track of children’s intake of sweets and snack foods, limiting availability of unhealthy foods, modeling healthy eating habits), and autonomy support (e.g., telling children that healthy foods taste good, encouraging them to eat a variety of foods) [1, 2].

Evidence consistently shows that both mothers’ and fathers’ food parenting practices play a vital role in shaping children’s eating behaviors [1]. Structure- and autonomy-based food parenting practices, considered supportive parenting practices, have been linked to healthier habits in children, including improved food choices and greater fruit and vegetable intake [3–6]. In contrast, use of coercive control has been shown to foster children’s preferences for nutrient-poor and energy-dense foods [5].

The vast majority of studies on food parenting practices [7, 8] and childhood obesity prevention [9, 10] have focused on mothers, leaving fathers understudied, even though fathers’ engagement is important within the family context for children’s development and eating behaviors [11]. Grounded in the Life Course Perspective [12] and the Men’s Preconception Health paradigm [13], fathers’ preconception social, and psychological factors may be linked to health trajectories and behaviors across generations [12]. In this context, recent research indicates that fathers’ diet behaviors during adolescence are associated with reproductive outcomes and diet behaviors during fatherhood [13, 14].

What is not known is whether diet behaviors established in adolescence may be associated with how males later engage with their children around food. Dietary patterns begin to take root during adolescence, a formative period in which individuals develop food preferences, attitudes, and behaviors that influence future eating practices and downstream health outcomes [15, 16]. These dietary habits formed during adolescence may have lasting implications for fathers’ approach to feeding and food-related decisions as parents.

Identifying these critical periods in fathers’ lives that shape their food parenting practices can help inform strategies to support healthier child and family food environments. Therefore, this study examined whether fathers’ dietary patterns during adolescence are associated with their later food parenting practices. We hypothesized that fathers who followed healthier dietary patterns during adolescence would be more likely to engage in supportive food parenting practices.

## Methods

### Study population

This study utilized data from Fathers & Family Study (F&F) [60], a father-based cohort that recruited participants from an ongoing longitudinal study in the United States, the Growing Up Today Study (GUTS) [17, 18]. GUTS was originally launched in 1996 to investigate health behaviors over the life course [17, 18]. The original cohort (GUTS I) included 16 882 boys and girls aged 9 to 14 years, and a second cohort (GUTS II) was established in 2004, adding 10 923 participants aged 10 to 17 years [17, 18].

In 2021, male participants from GUTS I and II were invited to join [60]. Invitations were sent via email (*n* = 7637) and postal mail (*n* = 10,979), directing individuals to complete an online eligibility screener. To qualify, participants had to identify as biological, adoptive, or social fathers of a child aged 1 to 6 years, reside in the U.S., and live with the child for more than half of the time. Of the 1391 men who completed the screener, 756 met the eligibility criteria, and 750 consented to participate and were enrolled in the study.

**Fig. 1:**
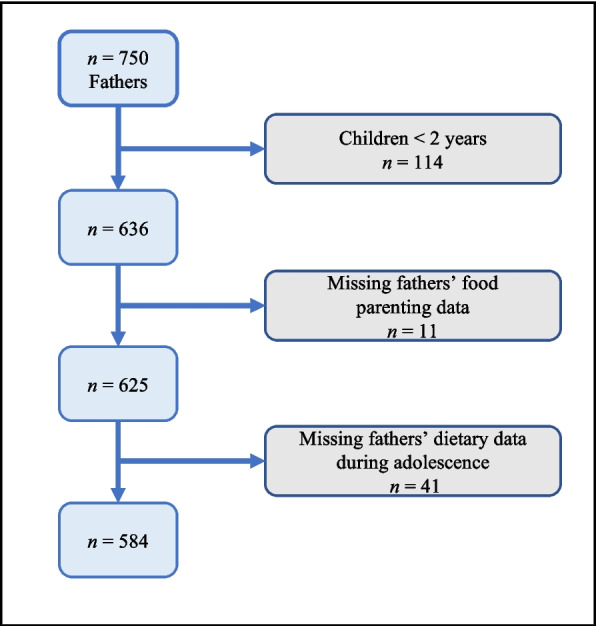
Flowchart of participant selection. *n*: number of participants

The present study utilized dietary and family meals data collected from the GUTS I and II cohorts across multiple survey waves (yearly from 1996–1999, and 2001, 2004, 2006, 2008, 2011), along with sociodemographic and food parenting data from the F&F collected in 2021–2022. The analytic sample for this study included F&F participant who reported having a child aged 2 years or older *n* = 636, as the food parenting practices measure has not been validated for use with parents of children under 2. Participants who completed fewer than 50% of items within a food parenting domain were excluded from the analytic sample (*n* = 11). For eight participants (1.4%) who completed at least 50% of items within a domain but had some missing responses, missing values were imputed using the mean of the completed items, assuming this level of item completion provides a reasonable approximation of the individual’s domain score. We further excluded individuals with less than two dietary assessments collected during adolescence (ages 10–18 years) (*n* = 41), resulting in a final analytic sample of 584 fathers, with a total of 2611 adolescent dietary assessments collected during adolescence (Fig. 1). Participants contributed between two and six dietary assessments: 14.9% contributed two, 11.3% contributed three, 16.1% contributed four, 27.2% contributed five, and 30.5% contributed six assessments. The duration of repeated assessments ranged from 2 to 7 years; 77.5% of fathers contributed dietary data spanning at least 4 adolescent years.

### Exposure

Diet quality patterns during adolescence (ages 10–18 years) assessed through the Healthy Eating Index (HEI-2020) were the primary exposure [19]. The HEI-2020 measures adhere to *The Dietary Guidelines for Americans, 2020–2025* [19, 20]. To characterize these patterns, we used fathers’ historical dietary intake self-reported during adolescence in the GUTS I and GUTS II surveys, assessed with the Youth/Adolescent Food Frequency Questionnaire (YAQ) between 1996 and 2011 [21–23]. Across GUTS surveys, the YAQ varied in length (from 28 to over 100 items): longer versions disaggregated broad food categories into specific subitems (e.g., different types of milk or cheese), whereas shorter versions retained aggregated categories [23]. Despite this variation in granularity, both versions capture the major food groups required to compute HEI-2020 components, supporting comparability across years [21–23]. Following YAQ documentation, categorical frequency of consumption data (e.g., times per week) were standardized to daily intake values using the midpoints of the response ranges, then converted to grams by applying standard serving weights for each food item [21–23]. In line with prior GUTS studies [24–26], we considered participants’ responses to reflect their actual intake and analyzed the data as provided, without applying additional exclusions based on the completeness of the YAQ section. Participants who left items blank were not excluded, as blanks were interpreted as non-consumption rather than missing responses. This approach is standard analytic practice for this YAQ and is supported by validation and methodological studies [27, 28] indicating that most omitted items correspond to foods consumed never or rarely, making zero intake a reasonable approximation when deriving dietary patterns.

To classify each participant’s dietary assessments according to the HEI-2020, each food item was mapped to a corresponding Food and Nutrient Database for Dietary Studies (FNDDS) food code from the 2017–2018 cycle [19, 29, 30]. This allowed for the decomposition of each food item into nutrient variables required for HEI-2020 scoring [19], which were then used to calculate overall HEI-2020 scores ranging from 0–100 for each dietary assessment using the *heiscore* package in R [31]. Using HEI-2020 guidelines [19], the longitudinal HEI-2020 scores were then categorized into grades as follows: F (< 60), D (60—< 70), C (70—< 80), B (80—< 90), and A (≥ 90).

Following the approach described by Vable et al. (2020), sequence analysis and hierarchical clustering were used to categorize participants’ diet quality patterns over time, based on all longitudinal HEI-2020 scores assessed between ages 10 and 18 years [32–34]. Using the R package *TraMineR*, each father’s HEI-2020 grades (A, B, C, D, or F) were converted into individual sequences representing their grade trajectories [33, 35]. Pairwise sequence dissimilarities among fathers’ dietary sequences during their adolescence were computed using Optimal Matching (OM), which determines the minimal transformation cost between sequences via insertions (adding a state into a sequence), deletions (removing a state from a sequence), and substitutions (replacing one state with another, e.g., changing “B” to “C”) [33, 36]. Each operation—substitution, insertion, and deletion—has an associated cost [33, 36]. A constant substitution cost matrix was applied, assigning equal costs to all substitutions [33, 36].

Hierarchical clustering with Ward’s linkage was performed on the resulting dissimilarity matrix to identify groups of participants with similar dietary trajectories [34]. To determine the optimal number of clusters, we used a combination of inertia plots and the elbow method, which together assess the trade-off between model complexity and explained variance [37, 38]. Specifically, we calculated the total within-cluster inertia, defined as the sum of squared dissimilarities (also referred to as the sum of squared errors, SSE) between each sequence and the representative sequence (medoid) of its assigned cluster [37]. As the number of clusters increases, this within-cluster SSE typically decreases, reflecting greater homogeneity within clusters [37]. The elbow method involves plotting the SSE across increasing numbers of clusters and identifying the point at which further increases result in only marginal reductions in SSE [38]. This inflection point—the “elbow”—marks the optimal number of clusters, balancing improved model fit with parsimony and helping avoid overfitting [38]. Based on this optimal number, fathers were assigned to clusters representing groups with similar dietary patterns.

### Outcomes

Fathers’ food parenting practices were assessed in the 2021–2022 F&F survey using 16 items adapted from the original 49-item Comprehensive Feeding Practices Questionnaire (CFPQ) [39]. Items were rated on a 4-point Likert scale ranging from 1 (strongly disagree) to 4 (strongly agree).

A recent study using F&F data identified 11 items that mapped onto three higher-order domains of food parenting practices: coercive control, structure, and autonomy support [2]. We used these 11 items to construct the same three domain-specific food parenting practice scores. Each score was derived from confirmatory factor analysis (CFA) loadings, then normalized and rescaled to range from 1 to 4, rounded to the nearest integer [40]. This transformation preserved the ordinal meaning, with 1 indicating the lowest and 4 the highest level of food parenting practice within each domain. Additional File 1 presents the items included in each domain along with their CFA loadings.

### Sociodemographic and confounder variables

Fathers reported sociodemographic characteristics in the 2021–2022 F&F survey, including their age, race/ethnicity and educational attainment along with their child’s age and sex. Race/ethnicity (categorized as Asian, Hispanic/Latino, Other, and White) and family meal frequency during adolescence were included as covariates in the regression models to minimize confounding based on prior literature [41–45]. Data on family meal frequency were retrieved from the GUTS I and II surveys, using a question that assessed how many times per week participants had dinner with their family [17, 18]. This measure was collected at multiple survey waves across adolescence; for the present analyses, we calculated each participant’s average family meal frequency across all available adolescent assessments to reflect typical exposure during adolescence. Although GUTS participants are children of nurses [17, 18], providing some homogeneity in maternal occupation, information on parental feeding practices or other household-level sociodemographic characteristics during participants’ adolescence (e.g., parental education, household income, or food security) was not available. Other covariates, such as child’s age and sex, were not included as adjustment variables because they occurred after the exposure period and, thus, cannot be confounders [46].

### Statistical analysis

To examine the association between fathers’ diet quality patterns during adolescence (identified using sequence analysis) and their food parenting practices, we fit a series of ordinal logistic regression models using the *MASS* package in R [35, 47]. Coercive control, structure, and autonomy support were analyzed as separate outcome variables with models adjusting for fathers’ race/ethnicity and adolescent family meal frequency. Results are reported on the odds ratios (OR) scale, along with 95% confidence intervals (CI) [48].

Additionally, we stratified by child’s sex (male/female) and age group (2–3 years and 4–6 years) to assess whether the association varied across these subgroups, who may have distinct eating behaviors [49]. The Brant test [50] was used to assess proportional odds in the fitted ordinal logistic regression models, and all analyses were performed using R v4.4.1 [35].

## Results

### Sample characteristics

The majority of fathers were between 35 and 40 years old (77.2%), identified as White (93.5%), and held at least a bachelor’s degree (88.7%). About half of children (54.1%) were male, and 63.4% were aged 2–3 years.

### Diet quality patterns during adolescence

**Fig. 2:**
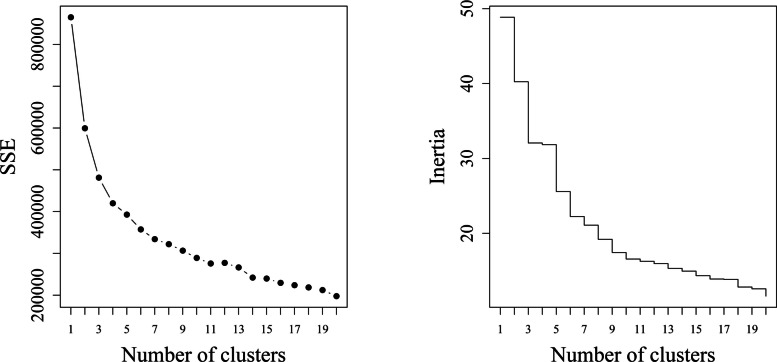
Determining the optimal number of clusters using the elbow method and inertia plot. SSE: sum of squared errors

**Fig. 3:**
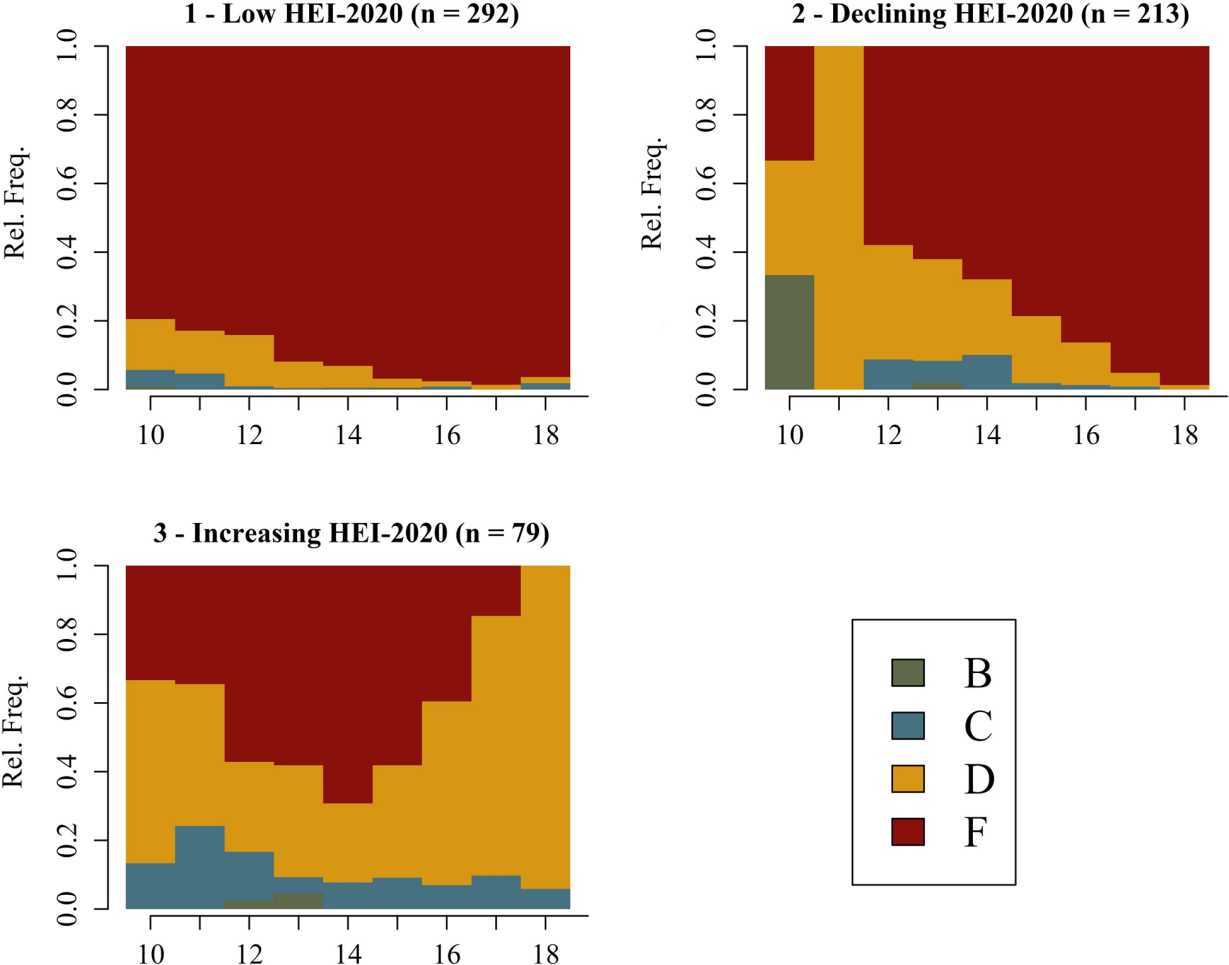
Clustering of fathers into three diet quality patterns based on their HEI-2020 scores during adolescence. n: Number of participants; Rel.Freq: Relative Frequency; HEI-2020: Healthy Eating Index-2020; B: 80 ≤ HEI-2020 score < 90; C: 70 ≤ HEI-2020 score < 80; D: 60 ≤ HEI-2020 score < 70; F: HEI-2020 score < 60

Sequence analysis and hierarchical clustering identified three clusters (Fig. 2) as the optimal solution for the diet quality patterns during adolescence (Fig. 3). The first pattern, labeled “Low HEI-2020,” primary consisted of fathers who consistently maintained the lowest HEI-2020 score (F) throughout adolescence (50.0%). The second, “Declining HEI-2020,” included fathers who started adolescence with higher HEI-2020 scores (B, C, D) but experienced a decline to the lowest score (D, F) by the end of adolescence (36.5%). Finally, the “Increasing HEI-2020” pattern consisted of fathers who improved their HEI-2020 scores during adolescence (13.5%). Fathers’ and children’s sociodemographic characteristics across these patterns are presented in Table 1.

**Table 1:** Fathers’ and children’s sociodemographic characteristics by fathers’ diet quality patterns during adolescence

	**Full Sample**	**Low** **HEI-2020**	**Declining** **HEI-2020**	**Increasing** **HEI-2020**
	*n* = 584	*n* = 292	*n* = 213	*n* = 79
	%	%	%	%
Father’s age (years)				
27 to 34	22.8	18.8	23.9	34.2
35 to 40	77.2	81.2	76.1	65.8
Father’s race/ethnicity				
Asian	2.4	1.0	3.8	3.8
Hispanic/Latino	1.5	1.7	1.9	0.0
Other	2.4	2.4	2.3	2.5
White	93.5	94.5	92.0	93.7
Not Reported	0.2	0.3	0.0	0.0
Father's educational level				
No college degree	11.0	12.3	9.4	10.1
Bachelor's degree	43.0	39.4	47.4	44.3
Postgraduate degree	45.7	47.9	42.7	45.6
Not Reported	0.3	0.3	0.5	0.0
Child’s sex				
Male	54.1	50.3	59.2	54.4
Female	45.9	49.7	40.8	45.6
Child’s age				
2−3 years old	63.4	64.7	60.1	67.1
4–6 years old	36.6	35.3	39.9	32.9

*HEI-2020* Healthy Eating Index-2020, *n* number of participants

Table 2 summarizes fathers’ diet quality scores for the entire sample and the three diet quality groups. These summarized results are for two time points during adolescence (first and last measurement) and therefore do not capture intermediate changes in diet quality, including temporary improvements followed by later declines. At the first assessment, 82.9% of participants in the Low HEI-2020 pattern were classified in the lowest HEI-2020 category (F), compared to 68.1% in the Declining HEI-2020 pattern and 51.9% in the Increasing HEI-2020 pattern. By the final assessment of adolescence, the proportion classified in category F increased to 96.6% among fathers in the Low HEI-2020 pattern and 97.7% in the Declining HEI-2020 pattern, while it dropped sharply to just 1.3% among fathers in the Increasing HEI-2020 pattern.

**Table 2:** Fathers’ adherence to HEI-2020 during adolescence across diet quality pattern groups

	**Full Sample**	**Low** **HEI-2020**	**Declining** **HEI-2020**	**Increasing** **HEI-2020**
	*n* = 584	*n* = 292	*n* = 213	*n* = 79
	%	%	%	%
Adolescence: *First assessment*				
A/B/C (HEI-2020 ≥ 70)	5.1	3.1	5.2	12.7
D (60 ≥ HEI-2020 < 70)	21.6	14.0	26.8	35.4
F (HEI-2020 < 60)	73.3	82.9	68.1	51.9
Adolescence:* Last assessment*				
A/B/C (HEI-2020 ≥ 70)	1.9	1.0	0.0	10.1
D (60 ≥ HEI-2020 < 70)	14.0	2.4	2.3	88.6
F (HEI-2020 < 60)	84.1	96.6	97.7	1.3

This table presents only the first and last assessments; intermediate changes in diet quality—including temporary improvements followed by later declines—are not shown

First assessment: The father's HEI-2020 classification at the first diet evaluation between ages 10 and 18;

Last assessment: The father's HEI-2020 classification at the last diet evaluation between ages 10 and 18

*n*: number of participants;

*HEI-2020* Healthy Eating Index-2020

### Diet quality patterns during adolescence and fathers’ food parenting practices

Results from the ordinal logistic regression examining the association between fathers’ diet quality patterns during adolescence and their food parenting practices, adjusted for family meal frequency and race/ethnicity (Table 3), revealed distinct associations across the three parenting domains. Compared to fathers in the Low HEI-2020 pattern, fathers in the Increasing HEI-2020 pattern had significantly lower odds of using coercive control (OR = 0.57, 95% CI 0.33–0.91) and higher odds of using structure-based food parenting practices (OR = 1.93, 95% CI 1.18–3.18). Food parenting practices did not differ significantly between fathers in the Low HEI-2020 and Decreasing HEI-2020 group. Additionally, no significant associations were observed between fathers’ diet quality patterns during adolescence and autonomy support food parenting practices.

**Table 3:** Adjusted associations between fathers’ diet quality trajectories during adolescence and their food parenting

	**OR**	**95% CI**	***p*-value**
Coercive Control			
Low HEI-2020	1.00	-	-
Declining HEI-2020	0.78	0.56–1.08	0.137
Increasing HEI-2020	0.57	0.36–0.91	0.019
Structure			
Low HEI-2020	1.00	-	-
Declining HEI-2020	1.17	0.82–1.67	0.381
Increasing HEI-2020	1.93	1.18–3.18	0.010
Autonomy Support			
Low HEI-2020	1.00	-	-
Declining HEI-2020	1.00	0.69–1.44	0.983
Increasing HEI-2020	0.80	0.49–1.33	0.394

Estimates derived from ordinal logistic regression models, with father’s race/ethnicity and family meals frequency during adolescence included as confounders in the analysis

*HEI-2020* Healthy Eating Index-2020, *OR* Odds ratio. *95%CI* 95% confidence interval

In the stratified analysis by child sex and age (Additional Files 2 and 3), trends were directionally similar. Fathers in the Increasing HEI-2020 pattern reported significantly lower coercive control and higher structure-based practices among girls and younger children; these associations were not significant for boys or older children. Across all models, results from Brant tests did not reveal significant evidence (i.e. all *p*-values > 0.05) of non-proportionality in ORs across outcome levels.

## Discussion

To our knowledge, this is the first study to examine the associations between fathers’ diet quality patterns during adolescence and their food parenting practices during fatherhood. To date, no studies have examined this association among mothers either. Prior work [51] has largely relied on retrospective designs, assessing connections between parents’ recollections of how they were fed during childhood and their current food parenting practices, rather than examining adolescent diet quality as an upstream predictor of later food parenting practices.

In this study, fathers who had increasing HEI-2020 scores across adolescence, compared with those with Low HEI-2020 scores, had nearly double the odds of reporting high structure and about 40% lower odds of high coercive control with their young children. In stratified analyses by child sex and age group, associations were generally consistent in direction; however, statistical significance was observed primarily among girls and younger children, with attenuated and less precise estimates among boys and older children, likely reflecting reduced statistical power following stratification rather than meaningful effect heterogeneity. No association was observed between fathers’ diet quality patterns during adolescence and use of autonomy support food parenting practices. These findings reinforce the Life Course Perspective [12] by suggesting that adolescence is not only a critical window for establishing long-lasting healthy eating behaviors, but also a period in which improvements in diet quality can support health-promoting food parenting practices during fatherhood.

The link between improving HEI-2020 scores during adolescence and supportive food parenting practices in fatherhood may reflect two complementary pathways. First, efforts to improve diet quality in adolescence may require use and development of self-regulation skills [52]. Importantly, adolescent eating patterns reflect a transitional developmental period where the strong influence of family and parents seen in early adolescence gradually diminishes as individuals transition to middle/late adolescence and gain greater autonomy over food choices and eating contexts, particularly outside the home [53]. Fathers may later draw on these skills when managing feeding responsibilities, enabling them to establish structured feeding environments and avoid controlling feeding tactics in with their young children [52]. Second, improvements in diet quality during adolescence may result from earlier exposure to supportive food parenting practices within boys’ families of origin [54]. Research indicates that parenting practices, especially those related to feeding, are often transmitted across generations, with individuals tending to adopt similar practices with their own children to which they were exposed in childhood [51, 55, 56]. These intergenerational patterns suggest that boys who are exposed to supportive and health-promoting food environments may not only benefit personally but also be more likely to recreate such environments as future fathers [54, 57, 58]. However, because we did not have direct measures of the food parenting practices to which fathers were exposed during childhood, these intergenerational pathways should be interpreted as potential explanatory mechanisms rather than tested components of the present analyses. At the same time, both adolescent dietary patterns and later use of specific food-parenting practices may index a broader interest in nutrition—and may be shaped by how nutrition is marketed to boys and young men—so gender-inclusive, culturally responsive initiatives are warranted within pre-conception health strategies [13].

There are two potential reasons why we did not observe associations between fathers’ diet quality patterns during adolescence and autonomy support food parenting practices in fatherhood. First, we only used two items to assess fathers’ autonomy support food parenting practices, and these items may not fully capture the multi-dimensional elements of autonomy support food parenting practices [2]. Second, fathers in our study highly rated the two autonomy support items, limiting data variability to detect meaningful associations [2]. Future studies should use more new nuanced autonomy support measurements to explore its association with diet quality patterns across adolescence.

This study presents several key strengths. Foremost is its novel prospective longitudinal data and focus on diet quality patterns during adolescence as predictors of fathers’ food parenting practices, a perspective that remains underexplored in the literature. By leveraging longitudinal data collected at multiple time points between ages 10 and 18, we were able to capture distinct dietary patterns during this formative period, which is a rare opportunity given the scarcity of such longitudinal dietary data [15, 16]. This approach provided unique insights into how early nutritional habits may shape the feeding environments of the next generation.

Another strength is the use of the HEI-2020 scores to assess diet quality [19]. It directly indexes adherence to *The Dietary Guidelines for Americans, 2020–2025*, making findings policy-relevant and interpretable [19, 20]. Although the YAQ length varied substantially across waves (≈28 to > 100 items), HEI-2020 can still be computed and compared because it is derived from harmonized food-group components and emphasizes component densities rather than absolute intake [19, 23]. In addition, our longitudinal sequence analysis with hierarchical clustering focuses on relative, within-person change, which reduces sensitivity to variation in item coverage and reveals distinct diet-quality trajectories that capture the heterogeneity and complexity of adolescent eating [33, 34].

Despite its strengths, this study also had limitations that should be considered. First, fathers in our study were predominantly White and highly educated, potentially limiting our findings’ generalizability to other populations. In addition, the possibility of self-selection bias exists, as fathers who chose to participate in F&F may have been more health-conscious or engaged in more supportive parenting behaviors [59]. Future research should include more diverse and representative samples across racial/ethnic and socioeconomic backgrounds and varying levels of health motivation to improve generalizability and more accurately capture population-level patterns. Second, both dietary intakes and food parenting practices were self-reported by fathers and may be subject to measurement error or social desirability bias, which likely reduced the precision of our estimates [59]. Third, our data did not allow us to examine the links between fathers with other diet quality patterns across adolescence, such as consistently high HEI-2020 scores, and food parenting practices during fatherhood, warranting further investigations in future studies. Fourth, unmeasured confounders related to fathers’ early-life context may have influenced the observed associations. In particular, we lacked direct measures of the food parenting practices to which fathers were exposed during their own childhood, as well as detailed household-level sociodemographic information beyond maternal occupation, limiting our ability to assess more complex models of multiple potential pathways underlying the intergenerational transmission of dietary behaviors. Additionally, fathers’ current family context—such as partners’ (maternal) food parenting practices, relationship status, and number of children in the household—may also shape paternal food parenting behaviors, potentially intersecting with their dietary histories. Future research may include richer sets of potential confounding factors or more rigorous designs to strengthen causal inference and explore multiple pathways underlying the intergenerational transmission of dietary behaviors.

## Conclusions

In summary, this study highlights prospective associations between fathers’ diet quality patterns during adolescence and their later food parenting practices with their young children. Fathers who improved their diet quality during adolescence were more likely to report lower use of coercive control and higher use of structure-based food parenting practices compared to those with consistently poor diet quality. These findings suggest that adolescence may represent an important period for nutrition interventions among boys, which have traditionally been linked to men’s own health but may also be associated with food parenting practices and the dietary environments they provide for their children.

## Supplementary Information

Additional file 1. Confirmatory Factor Analysis of food parenting practices. F1: Factor 1; F2: Factor 2; F3: Factor 3.

Additional file 2. Adjusted associations between fathers' diet quality patterns during adolescence and their food parenting strategies, stratified by child's sex.

Additional file 3. Adjusted associations between fathers' diet quality patterns during adolescence and their food parenting strategies, stratified by child's age group.

## Data Availability

The data underlying this article will be shared on reasonable request to the corresponding and last author.

## Abbreviations

CFA: Confirmatory Factor Analysis
CFPQ: Comprehensive Feeding Practices Questionnaire
CI: Confidence Interval
F&F: Fathers & Families (Study)
FNDDS: Food and Nutrient Database for Dietary Studies
GUTS: Growing Up Today Study
HEI-2020: Healthy Eating Index 2020
IRB: Institutional Review Board
OM: Optimal Matching
OR: Odds Ratio
SD: Standard Deviation
YAQ: Youth/Adolescent Food Frequency Questionnaire
