# Supplementary figures and images for: Men’s preconception diet quality patterns predict supportive food parenting practices: evidence from a longitudinal cohort study
Source: Int J Behav Nutr Phys Act. 2026 May 1;23:66. doi: 10.1186/s12966-026-01914-z (PMC13321645; doi:10.1186/s12966-026-01914-z)

## Coercive Control

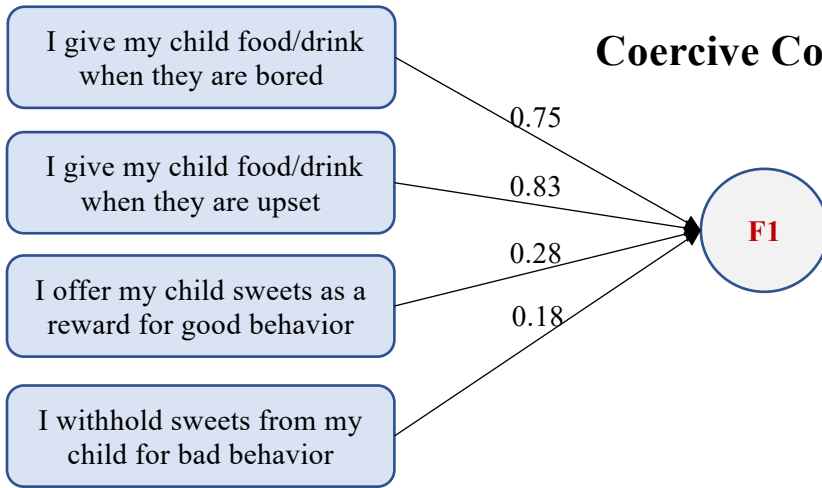

## Structure

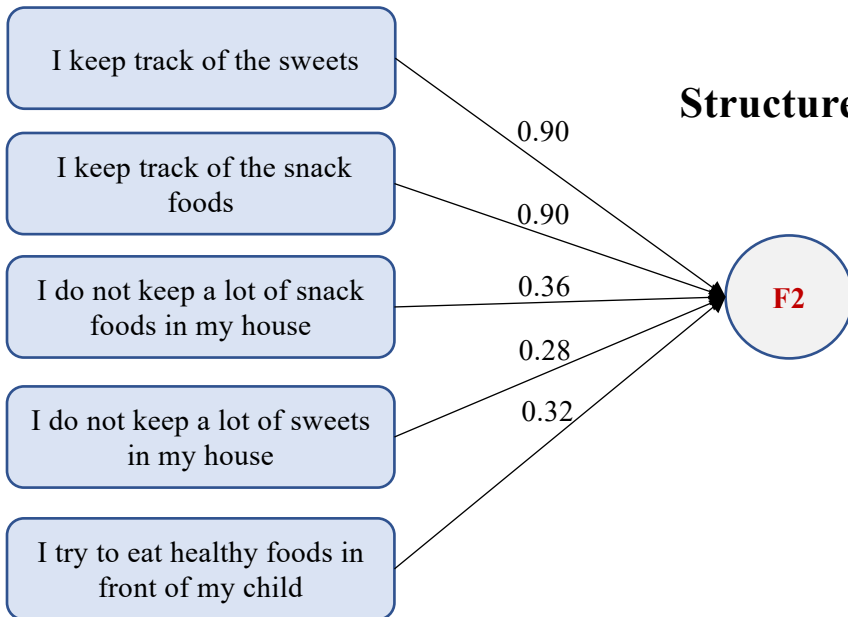

## Autonomy Support

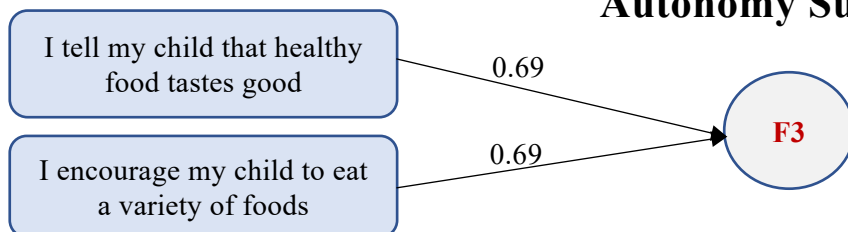

Supplement: Supplementary file 1 — Additional file 1. Confirmatory Factor Analysis of food parenting practices. F1: Factor 1; F2: Factor 2; F3: Factor 3. [file 12966_2026_1914_MOESM1_ESM.pdf]
