# Supplementary material for: Men’s preconception diet quality patterns predict supportive food parenting practices: evidence from a longitudinal cohort study
Source: Int J Behav Nutr Phys Act. 2026 May 1;23:66. doi: 10.1186/s12966-026-01914-z (PMC13321645; doi:10.1186/s12966-026-01914-z)
Supplement: Supplementary file 2 — Additional file 2. Adjusted associations between fathers' diet quality patterns during adolescence and their food parenting strategies, stratified by child's sex. [file 12966_2026_1914_MOESM2_ESM.docx]

**Additional File 2**: Adjusted associations between fathers' diet quality patterns during adolescence and their food parenting strategies, stratified by child's sex.

|  | **Male** (*n* = 316) | | | **Female** (*n* = 268) | | |
| --- | --- | --- | --- | --- | --- | --- |
| Diet quality patterns | **OR** | **95%CI** | **p-value** | **OR** | **95%CI** | **p-value** |
| Coercive Control |  |  |  |  |  |  |
| Low HEI-2020 | 1.00 | - | - | 1.00 | - | - |
| Declining HEI-2020 | 0.86 | 0.55-1.34 | 0.508 | 0.68 | 0.41-1.12 | 0.132 |
| Increasing HEI-2020 | 0.68 | 0.37-1.26 | 0.220 | 0.46 | 0.22-0.94 | 0.036 |
|  |  |  |  |  |  |  |
| Structure |  |  |  |  |  |  |
| Low HEI-2020 | 1.00 | - | - | 1.00 | - | - |
| Declining HEI-2020 | 1.09 | 0.68-1.75 | 0.726 | 1.32 | 0.77-2.28 | 0.312 |
| Increasing HEI-2020 | 1.89 | 0.95-3.77 | 0.069 | 2.01 | 0.97-4.21 | 0.062 |
|  |  |  |  |  |  |  |
| Autonomy Support |  |  |  |  |  |  |
| Low HEI-2020 | 1.00 | - | - | 1.00 | - | - |
| Declining HEI-2020 | 1.35 | 0.82-2.22 | 0.227 | 0.71 | 0.40-1.26 | 0.237 |
| Increasing HEI-2020 | 1.23 | 0.62-2.45 | 0.562 | 0.47 | 0.22-1.01 | 0.050 |

HEI-2020: Healthy Eating Index-2020;

OR: Odds ratio;

95%CI: 95% confidence interval;

*n*: number of participants;

Note: Estimates derived from ordinal logistic regression models, with father’s race/ethnicity and family meals frequency during adolescence included as confounders in the analysis.
