# Supplementary material for: Men’s preconception diet quality patterns predict supportive food parenting practices: evidence from a longitudinal cohort study
Source: Int J Behav Nutr Phys Act. 2026 May 1;23:66. doi: 10.1186/s12966-026-01914-z (PMC13321645; doi:10.1186/s12966-026-01914-z)
Supplement: Supplementary file 3 — Additional file 3. Adjusted associations between fathers' diet quality patterns during adolescence and their food parenting strategies, stratified by child's age group. [file 12966_2026_1914_MOESM3_ESM.docx]

**Additional File 3:** Adjusted associations between fathers' diet quality patterns during adolescence and their food parenting strategies, stratified by child's age group.

|  | ***Child's age: 2-3y*** (*n* = 370) | | | ***Child's age: 4-6y*** (*n* = 214) | | |
| --- | --- | --- | --- | --- | --- | --- |
| Diet quality patterns | **OR** | **95%CI** | **p-value** | **OR** | **95%CI** | **p-value** |
| Coercive Control |  |  |  |  |  |  |
| Low HEI-2020 | 1.00 | - | - | 1.00 | - | - |
| Declining HEI-2020 | 0.63 | 0.41-0.97 | 0.035 | 1.07 | 0.63-1.84 | 0.799 |
| Increasing HEI-2020 | 0.49 | 0.27-0.86 | 0.014 | 0.75 | 0.33-1.69 | 0.487 |
|  |  |  |  |  |  |  |
| Structure |  |  |  |  |  |  |
| Low HEI-2020 | 1.00 | - | - | 1.00 | - | - |
| Declining HEI-2020 | 1.29 | 0.82-2.02 | 0.270 | 0.99 | 0.55-1.81 | 0.986 |
| Increasing HEI-2020 | 1.88 | 1.03-3.47 | 0.041 | 2.01 | 0.84-4.89 | 0.120 |
|  |  |  |  |  |  |  |
| Autonomy Support |  |  |  |  |  |  |
| Low HEI-2020 | 1.00 | - | - | 1.00 | - | - |
| Declining HEI-2020 | 0.92 | 0.57-1.49 | 0.727 | 1.22 | 0.68-2.19 | 0.513 |
| Increasing HEI-2020 | 0.60 | 0.32-1.11 | 0.099 | 1.37 | 0.57-3.46 | 0.492 |

HEI-2020: Healthy Eating Index-2020;

OR: Odds ratio;

95%CI: 95% confidence interval;

*n*: number of participants;

Note: Estimates derived from ordinal logistic regression models, with father’s race/ethnicity and family meals frequency during adolescence included as confounders in the analysis.
